# Elevated serum soluble α3(IV)NC1 correlates with kidney injury and worse outcome in patients with anti-glomerular basement membrane disease

**DOI:** 10.3389/fimmu.2026.1728059

**Published:** 2026-02-20

**Authors:** Huang Kuang, Hong-shan Wan, Zhao Cui, Ming-hui Zhao, Xiao-yu Jia

**Affiliations:** 1 Renal Division, Peking University First Hospital, Beijing, China; 2 Institute of Nephrology, Peking University, Beijing, China; 3 Key Laboratory of Renal Disease, Ministry of Health of China, Beijing, China; 4 Key Laboratory of CKD Prevention and Treatment, Ministry of Education of China, Beijing, China

**Keywords:** anti-glomerular basement membrane disease, clinical significance, outcome, soluble antigen, α3(IV)NC1

## Abstract

**Background:**

Anti-glomerular basement membrane (GBM) disease is the most severe form of autoimmune kidney diseases characterized by pathogenic autoantibodies against GBM component, α3(IV)NC1. However, the status of circulating soluble α3(IV)NC1 (also known as tumstatin) in patients with anti-GBM disease and its clinical relevance remain unclear. The aim of this study was to investigate the serum soluble α3(IV)NC1 level [ss-α3(IV)NC1] in patients with anti-GBM disease, and to analyze its association to clinical characteristics.

**Methods:**

Seventy patients with anti-GBM disease and 30 healthy individuals were enrolled. Ss-α3(IV)NC1 was measured by enzyme-linked immunosorbent assay. The level was compared and analyzed with clinical-pathological features and kidney outcome, and the statistical significance was determined.

**Results:**

Ss-α3(IV)NC1 concentrations were elevated in 41 (58.6%) patients with anti-GBM disease and 0 (0.0%) healthy individuals. They were significantly higher in patients with anti-GBM disease (18.32 ± 9.56 ng/mL) than those in healthy individuals (5.84 ± 2.78 ng/mL) (*P* < 0.001). Ss-α3(IV)NC1 correlated with age (*r* = 0.385, *P* = 0.001), serum creatinine (*r* = 0.286, *P* = 0.016), eGFR (*r* = -0.304, *P* = 0.010), crescent percentage (*r* = 0.339, *P* = 0.030), and normal glomeruli percentage (*r* = -0.354, *P* = 0.023). Kaplan–Meier analysis revealed that patients with elevated ss-α3(IV)NC1 had a worse kidney outcome than those with normal level (*P* = 0.011).

**Conclusions:**

The ss-α3(IV)NC1 was abnormally elevated in patients with anti-GBM disease. Elevated ss-α3(IV)NC1 was associated with the disease severity in anti-GBM disease. The role of these elevated levels warrants further investigation.

## Introduction

Anti-glomerular basement membrane (GBM) disease is an autoimmune disorder mediated by circulating autoantibodies directed against the GBM and alveolar basement membrane (ABM) (1, 2). It usually presents as rapidly progressive glomerulonephritis with or without pulmonary hemorrhage (3). The diagnosis is typically made based on the presence of linear IgG deposition along the GBM on kidney biopsy, and the detection of circulating anti-GBM antibodies. The primary target antigen recognized by anti-GBM antibodies is the non-collagenous domain 1 (NC1) of the α3 chain of type IV collagen [α3(IV)NC1] (4). Two conformation-dependent epitopes have been mapped to the amino acid residues 17–31 and 127–141 on α3(IV)NC1, also known as E_A_ and E_B_ respectively (5).

Under physiological conditions, these two epitopes of α3(IV)NC1 are thought to be sequestered within the hexameric structure of the GBM (6). However, α3(IV)NC1 is not a strictly “sequestered” autoantigen within the basement membrane. During basement membrane synthesis and turnover, it could be released into the circulation, where it serves vital physiological functions (7). Previous studies have shown that soluble form of α3(IV)NC1 (also known as tumstatin) has an anti-tumor effect by inhibiting capillary endothelial cell proliferation and angiogenesis (8). Due to the restricted distribution of the α3 chain of type IV collagen (COL4A3) in human basement membranes, the GBM and ABM serve as the primary source of serum soluble α3(IV)NC1 [ss-α3(IV)NC1]. Such a distribution is consistent with the understanding that anti-GBM disease is an organ-specific disorder that only affects the kidneys and/or lungs (3). Ss-α3(IV)NC1 is derived from the degradation of basement membranes containing COL4A3 protein by matrix metalloproteinases (MMPs), and is present at low level under physiological conditions (7, 9). Previous studies revealed that ss-α3(IV)NC1 was abnormally elevated and correlated with poor outcome in patients with lung cancer (10, 11). In anti-GBM disease, MMPs are also thought to be involved in cleaving GBM components, thereby exacerbating kidney pathology (12). However, it is unclear whether the ss-α3(IV)NC1 produced in this cleavage process plays a role in the disease progression.

Therefore, in the study, we measured the soluble α3(IV)NC1 level in serum of patients with anti-GBM disease, compared the level with those of healthy individuals, and analyzed the association between ss-α3(IV)NC1 level, clinical-pathological characteristics, and kidney outcome of anti-GBM disease.

## Materials and methods

### Patients and controls

Seventy sequential patients with antibody-positive anti-GBM disease, diagnosed in Peking University First Hospital from July 2015 to July 2022 were included in this study. The clinical and pathological data of all patients were collected at the time of diagnosis as well as during follow-up. Seven patients with negative anti-GBM antibodies and incomplete clinical data were excluded. Anti-GBM antibodies were measured at the time of disease onset by enzyme-linked immunosorbent assay (ELISA) (Euroimmun, Lubeck, Germany; positive cut-off value was set as > 20 RU/mL). Antigen-specific ANCA (myeloperoxidase or proteinase 3) were also measured in sera using a commercial ELISA kit (Euroimmun, Lubeck, Germany). The crescent percentage was defined as a fraction of glomeruli with crescent formation divided by the total number of glomeruli on each kidney biopsy. Normal glomeruli were defined as glomeruli that did not exhibit glomerulosclerosis, crescents, or fibrinoid necrosis (13). To evaluate kidney outcome, the primary endpoint was set as end-stage kidney disease (ESKD), defined as dependence on dialysis for more than 3 months and up to last follow-up. Complete histological data, available for 41 patients who underwent kidney biopsy, were assessed. The biopsy-proven anti-GBM disease was supported by the bright linear deposit of IgG fluorescence along the GBM, excluding other causes such as diabetes and fibrillary glomerulonephritis. The main reasons the remaining patients did not receive a kidney biopsy were: a high risk of bleeding, high levels of serum creatinine, and patient consent.

Age and gender matched control sera samples were obtained from 30 healthy individuals. All the serum samples were stored at -20°C until use. Informed consent was obtained for sampling blood. The research complied with the Declaration of Helsinki and was approved by the Ethics Committee of the same hospital.

### Quantitative detection of ss-α3(IV)NC1

The ss-α3(IV)NC1 was measured using a commercial ELISA kit (CUSABIO, China) following the manufacturer’s instructions. Antibody specific for α3(IV)NC1 was pre-coated onto a microplate. The standards ranged from 0.156 ng/mL to 10 ng/mL and serum samples diluted 1:10. The color development was stopped by adding the stop solution, and measured spectrophotometrically at 450 nm (Bio-Rad Laboratories, Philadelphia, PA).

### Statistical analysis

All statistical analyses were performed using R v4.4.1. The normality of continuous variables was assessed using the Shapiro-Wilk test. Continuous variables are presented as mean ± standard deviation (SD) or median with interquartile ranges, while categorical variables are presented as relative frequencies. The Student’s t-test or Mann-Whitney U test was used to examine differences for continuous variables, whereas the chi-square test or the Fisher exact test was used for categorical variables. Spearman’s correlation test was employed to investigate the correlations between ss-α3(IV)NC1 and clinical parameters. Factors predictive of kidney outcome were firstly evaluated by univariate Cox regression. Multivariate Cox regression analyses were conducted separately for both clinical (n =70) and pathological parameters (n = 41), given that the number of patients who underwent biopsy was limited. The performance of ss-α3(IV)NC1 for kidney outcome was evaluated by Kaplan-Meier analysis. A *P*-value < 0.05 was considered statistically significant.

## Results

### Characteristics of study participants

The major demographic, clinical and pathological data are shown in **Table 1**. Among the 70 patients with anti-GBM disease, 32 (45.7%) were male, the median age was 56.5 (43.0, 66.0) years, and 23 (32.9%) had a smoking history. Lung hemorrhage occurred in 14 (20.0%) patients. At diagnosis, the mean serum creatinine was 818.5 ± 470.9 μmol/L, corresponding to a median eGFR of 5.0 (3.5, 8.8) mL/min/1.73m². One third of the patients (24/70, 34.3%) had positive anti-neutrophil cytoplasmic antibodies (ANCA) and the median level of anti-GBM antibodies was 180.5 (113.0, 200.0) RU/mL. Forty (57.1%) of patients required renal replacement therapy (RRT) at the time of their admission. During last follow-up, 48 (68.6%) patients progressed to ESKD. Forty-one patients underwent kidney biopsies. Typical linear IgG deposition along the GBM was seen in 41 patient biopsies, and the median crescent percentage was 86.7 (75.0, 92.3).

**Table 1:** Baseline clinical characteristics of patients with anti-GBM disease.

Characteristic	Overall, *n* = 70
Male (n, %)	32 (45.7%)
Age (years)	56.5 (43.0, 66.0)
Hydrocarbon exposure (n, %)	7 (10.0%)
Smoking (n, %)	23 (32.9%)
Prodromal Infection (n, %)	47 (67.1%)
Lung hemorrhage (n, %)	14 (20.0%)
Oligoanuria (n, %)	38 (54.3%)
Gross hematuria (n, %)	39 (55.7%)
Anti-GBM antibodies (RU/mL)	180.5 (113.0, 200.0)
ANCA (n, %)	24 (34.3%)
Anti-MPO/PR3/both	22/2/0
Hemoglobin (g/L)	87.8 ± 19.3
Serum creatinine (μmol/L)	818.5 ± 470.9
eGFR (mL/min/1.73m²)	5.0 (3.5, 8.8)
Serum albumin (g/L)	30.6 ± 4.8
Proteinuria (g/day)	1.3 (0.8, 2.2)
Initial need for RRT (n, %)	40 (57.1%)
Plasma exchange (n, %)	67 (95.7%)
Prednisone (n, %)	69 (98.6%)
Cyclophosphamide (n, %)	53 (75.7%)
Methylprednisolone (n, %)	56 (80.0%)
Crescents (%)	86.7 (75.0, 92.3)
Normal glomeruli (%)	9.4 (0.0, 23.5)
ESKD (n, %)	48 (68.6%)

GBM, glomerular basement membrane; ANCA: anti-neutrophil cytoplasmic antibody; MPO, myeloperoxidase; PR3, proteinase 3; eGFR: estimated glomerular filtration rate; RRT: renal replacement therapy; ESKD, end-stage kidney disease.

### Ss-α3(IV)NC1 was elevated in patients with anti-GBM disease

**Figure 1:**
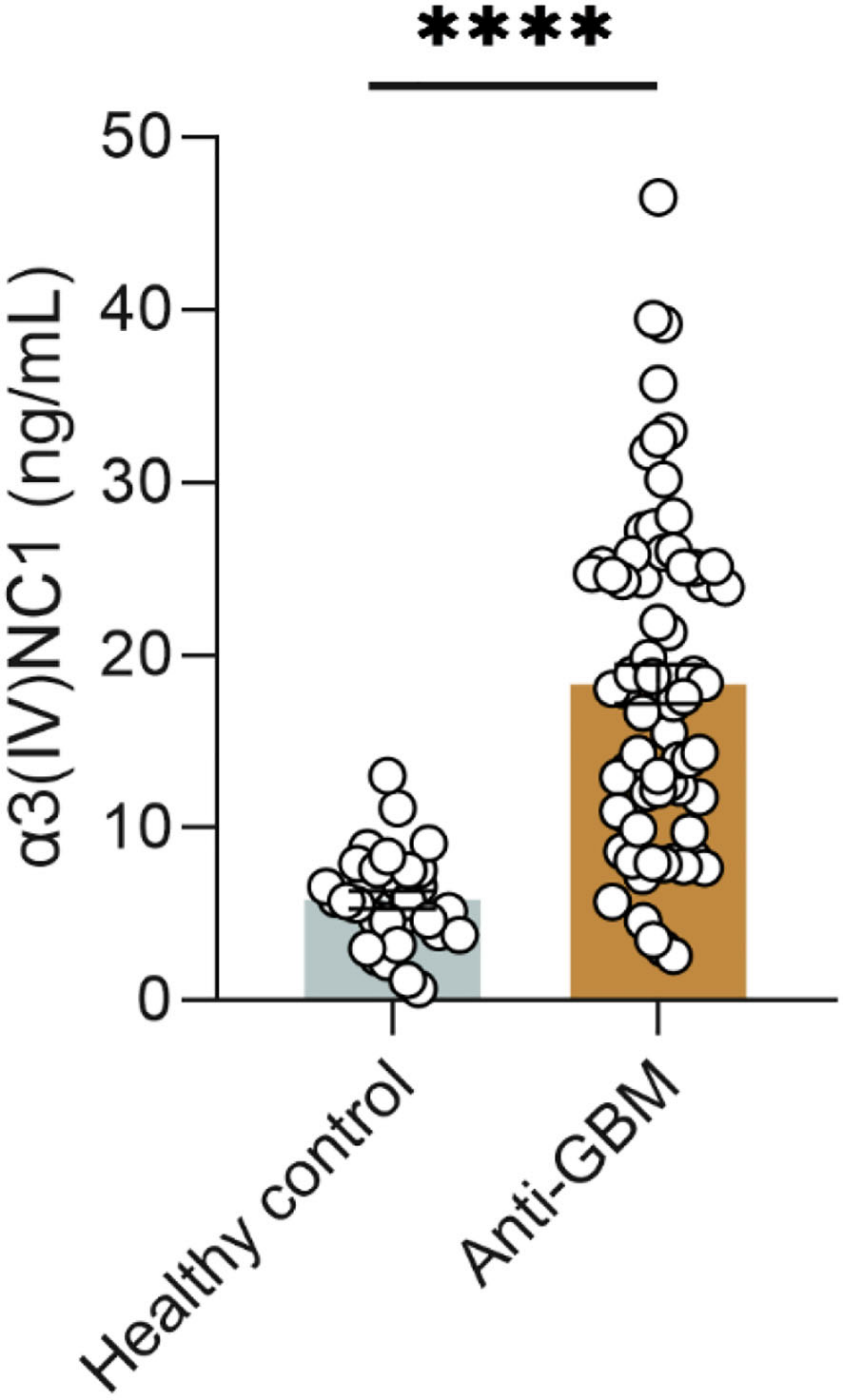
Ss-α3(IV)NC1 is elevated in patients with anti-GBM disease. Healthy control) *n* = 30, anti-GBM disease *n* = 70 per group. Data are expressed as mean ± SEM; statistical significance was determined by Mann-Whitney test. ****P-value < 0.0001

As shown in **Figure 1**, the ss-α3(IV)NC1 was significantly elevated in patients with anti-GBM disease (18.32 ± 9.56 ng/mL), compared with healthy individuals (5.84 ± 2.78 ng/mL) (*P* < 0.001). Using the mean + 3SD of ss-α3(IV)NC1 from healthy individuals as the cut-off value (14.024 ng/mL), the level was elevated in 41 (58.6%) patients with anti-GBM disease and 0 (0.0%) healthy individuals.

### Correlation between ss-α3(IV)NC1 and clinical-pathological features of anti-GBM disease

**Figure 2:**
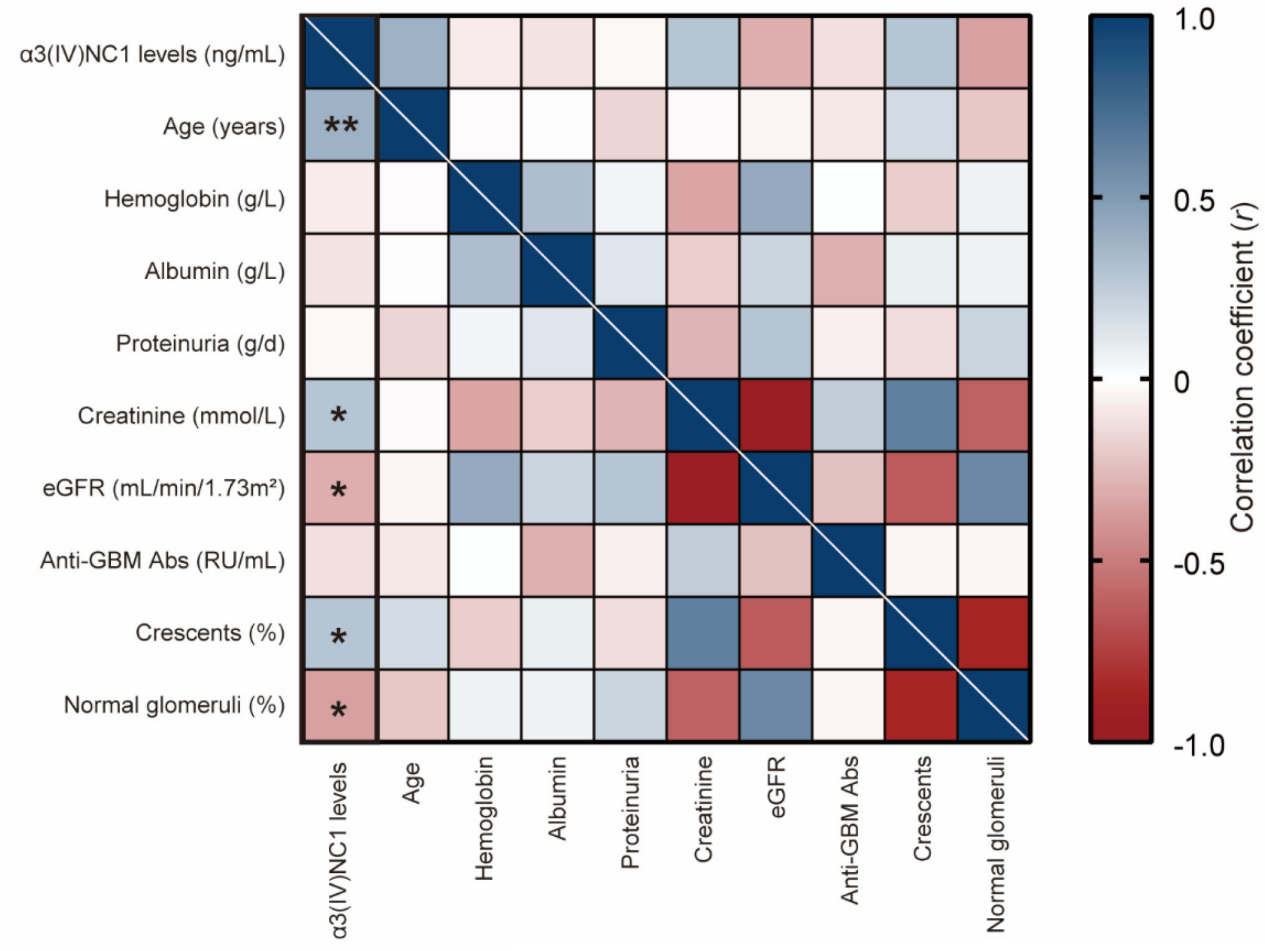
Ss-α3(IV)NC1 is associated with clinical and pathological characteristics in patients with anti-GBM disease. Association between ss-α3(IV)NC1 and parameters in patients with anti-GBM disease was shown by a heatmap reflecting *P*-value and Spearman’s correlation coefficient (*r*). Ss-α3(IV)NC1 was defined as a continuous variable here. eGFR: estimated glomerular filtration rate; Anti-GBM Abs, anti-glomerular basement membrane antibodies; *P-value < 0.05, **P-value < 0.01.

The correlations between ss-α3(IV)NC1 and clinical-pathological features of patients with anti-GBM disease are presented in **Figure 2**. An increased ss-α3(IV)NC1 was correlated with age (*r* = 0.385, *P* = 0.001), serum creatinine (*r* = 0.286, *P* = 0.016), and eGFR (*r* = -0.304, *P* = 0.010). Moreover, it was also positively correlated with the crescent percentage (*r* = 0.339, *P* = 0.030), and negatively correlated with normal glomeruli percentage (r = -0.354, *P* = 0.023).

### Features of patients with anti-GBM disease classified by ss-α3(IV)NC1

The above cut-off value of 14.024 ng/mL was determined to distinguish the group with elevated ss-α3(IV)NC1 level (*n* = 41) from the group with normal α3(IV)NC1 level (*n* = 29). The features of these two groups are shown in **Table 2**. The age at disease onset was higher in patients with elevated ss-α3(IV)NC1 compared with those with normal ss-α3(IV)NC1 (63 *versus* 45, *P* < 0.001). The frequency of gross hematuria was higher in the elevated ss-α3(IV)NC1 group than the normal ss-α3(IV)NC1 group (65.9 *versus* 41.4%, *P* = 0.042). ANCA positivity was observed in 43.9% of patients with elevated ss-α3(IV)NC1, which was higher than those with normal ss-α3(IV)NC1 level (20.7%, *P* = 0.044). The elevated ss-α3(IV)NC1 group showed increased serum creatinine (928.4 ± 459.3 *versus* 663.1 ± 449.7 μmol/L, *P* = 0.019) and low eGFR level (4.4 *versus* 8.7 mL/min/1.73m^2^, *P* = 0.005), compared with normal ss-α3(IV)NC1 group. After one year follow-up, 75.6% of patients in the elevated ss-α3(IV)NC1 group progressed to ESKD, which was higher than the normal ss-α3(IV)NC1 group (44.8%, *P* = 0.009). No statistically significant differences in gender, lung hemorrhage, level of anti-GBM antibodies, treatment regimens, and kidney pathology (crescent percentage and normal glomeruli percentage) were found between the two groups.

**Table 2:** Clinical characteristics of patients with anti-GBM disease according to ss-α3(IV)NC1.

Characteristic	Elevated (*n* = 41)	Normal (*n* = 29)	*P*-value
Male (n, %)	19 (46.3%)	13 (44.8%)	0.900
Age (years)	63 (51, 74)	45 (39, 62)	**< 0.001**
Hydrocarbon exposure (n, %)	(9.8%)	3 (10.3%)	> 0.999
Smoking (n, %)	15 (36.6%)	8 (27.6%)	0.430
Prodromal Infection (n, %)	25 (61.0%)	22 (75.9%)	0.191
Lung hemorrhage (n, %)	8 (19.5%)	6 (20.7%)	0.903
Oligoanuria (n, %)	25 (61.0%)	13 (44.8%)	0.182
Gross hematuria (n, %)	27 (65.9%)	12 (41.4%)	**0.042**
Anti-GBM antibodies (RU/mL)	162 (96, 200)	196 (128, 200)	0.201
ANCA positivity (n, %)	18 (43.9%)	6 (20.7%)	**0.044**
Hemoglobin (g/L)	85.8 ± 18.1	90.6 ± 20.9	0.311
Serum creatinine (μmol/L)	928.4 ± 459.3	663.1 ± 449.7	**0.019**
eGFR (mL/min/1.73m²)	4.4 (3.0, 6.2)	8.7 (4.2, 20.0)	**0.005**
Serum albumin (g/L)	30.5 ± 4.9	30.8 ± 4.9	0.819
Proteinuria (g/day)	1.3 (0.6, 1.9)	1.3 (0.8, 2.2)	0.965
Initial need for RRT (n, %)	26 (63.4%)	14 (48.3%)	0.207
Plasma exchange (n, %)	39 (95.1%)	28 (96.6%)	> 0.999
Prednisone (n, %)	40 (97.6%)	29 (100.0%)	> 0.999
Cyclophosphamide (n, %)	30 (73.2%)	23 (79.3%)	0.555
Methylprednisolone (n, %)	31 (75.6%)	25 (86.2%)	0.275
Crescents (%), *n* = 41	87.4 (75.7, 98.1)	86.7 (66.7, 91.7)	0.278
Normal glomeruli (%), *n* = 41	8.0 (0.0, 20.2)	10.0 (5.6, 33.3)	0.249
ESKD at 1 year (n, %)	31 (75.6%)	13 (44.8%)	**0.009**

GBM, glomerular basement membrane; ANCA, anti-neutrophil cytoplasmic antibody; eGFR, estimated glomerular filtration rate; RRT, renal replacement therapy; ESKD, end-stage kidney disease. Statistically significant differences were reported with a bold *P*-value.

**Figure 3:**
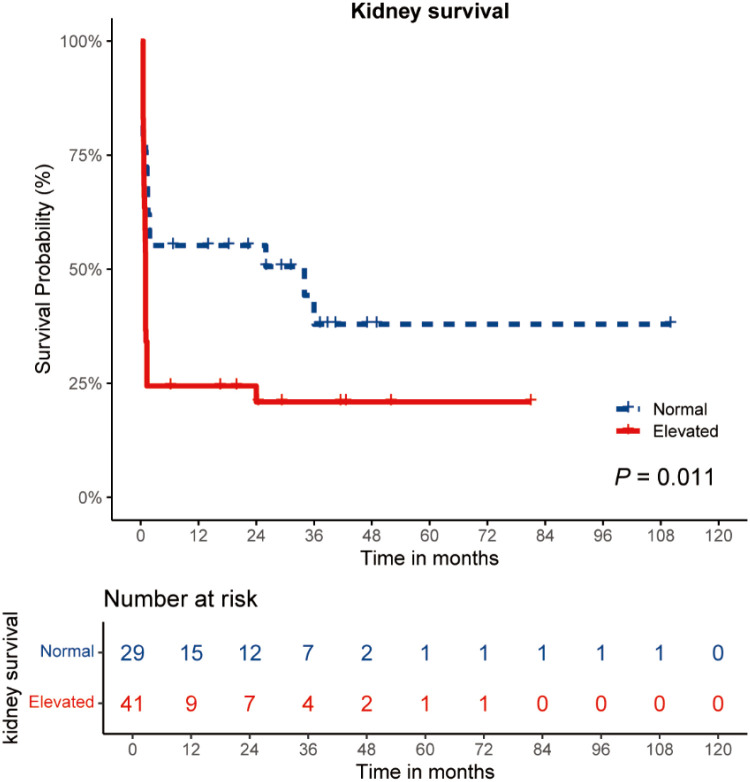
Curves of the cumulative incidence of end-stage kidney disease. Patients were divided into two groups according the ss-α3(IV)NC1. Comparison of survival curves was performed with log-rank test.

The Kaplan-Meier curve analysis was used to examine the kidney survival of the two groups of patients. It was shown that patients with elevated ss-α3(IV)NC1 level had worse kidney outcome compared to those with normal ss-α3(IV)NC1 level (*P* = 0.011) (**Figure 3**). The cumulative kidney survival rates at last time points in both groups were 20.9% and 37.9%, respectively.

### Risk factors for kidney survival in patients with anti-GBM disease

We identified risk factors associated with kidney survival in patients with anti-GBM disease using univariate Cox regression analysis (**Table 3**). Risk factors predicting kidney survival included oligoanuria (*P* < 0.001), ss-α3(IV)NC1 (*P* = 0.009), initial need for RRT (*P* < 0.001), serum creatinine level (*P* < 0.001), crescent percentage (*P* < 0.001), and normal glomeruli percentage (*P* < 0.001). On multivariate Cox analysis, the serum creatinine level and normal glomeruli percentage were independent factors predicting kidney outcome (*P* < 0.001 and *P* = 0.002, respectively; **Supplementary Table 1** and **Table 2**). Oligoanuria, ss-α3(IV)NC1, initial need for RRT, and crescent percentage did not have an additional influence on kidney outcome.

**Table 3:** Univariate Cox regression analysis for clinical-pathological indicators to predict kidney survival.

Predictors	*n*	HR (95% CI)	*P*-value
Age (years)	70	1.006 (0.988 to 1.025)	0.507
Gender (male)	70	1.331 (0.749 to 2.365)	0.330
Oligoanuria	70	2.830 (1.537 to 5.210)	**< 0.001**
Anti-GBM Abs (RU/mL)	70	1.000 (0.995 to 1.004)	0.906
ANCA positivity	70	1.196 (0.665 to 2.151)	0.549
ss-α3(IV)NC1 level (ng/mL)	70	1.039 (1.009 to 1.069)	**0.009**
Initial need for RRT	70	3.376 (1.801 to 6.327)	**< 0.001**
Serum creatinine (μmol/L)	70	1.002 (1.001 to 1.002)	**< 0.001**
Crescents (%)	41	1.094 (1.042 to 1.148)	**< 0.001**
Normal glomeruli (%)	41	0.893 (0.839 to 0.951)	**< 0.001**

Anti-GBM Abs, anti-glomerular basement membrane antibodies; ANCA, anti-neutrophil cytoplasmic antibodies; RRT, renal replacement therapy; HR, hazard ratio; 95% CI, 95% confidence interval. Statistically significant differences were reported with a bold *P*-value.

## Discussion

Anti-GBM disease is the most aggressive form of glomerulonephritis that leads to rapid decline of kidney function, and it is usually caused by pathogenic autoantibodies against GBM component α3(IV)NC1 (2). In this study, we demonstrated that ss-α3(IV)NC1 itself was significantly elevated in the circulation of patients with anti-GBM disease compared with healthy individuals. Key findings of our study are that increased ss-α3(IV)NC1 was correlated with kidney function injury and worse kidney outcome in patients with anti-GBM disease.

Our results showed that patients with elevated ss-α3(IV)NC1 level had higher serum creatinine and reduced eGFR levels than patients with a normal ss-α3(IV)NC1 level, suggesting that an elevated ss-α3(IV)NC1 level may be correlated with more severe disease activity. Moreover, a high frequency of ANCA positivity was observed in our cohort of patients with an elevated ss-α3(IV)NC1 level. This is clinically significant as ANCA-positive anti-GBM disease represents a distinct, often more severe phenotype of autoimmune injury. Given that elevated MMP-9—a key enzyme capable of cleaving α3(IV)NC1—has been documented in both serum and kidney tissues of patients with ANCA-associated vasculitis (AAV) (14–16), it is plausible that concurrent ANCA positivity acts as a confounding factor, directly contributing to the increase in our measured biomarker. Alternatively, the co-existence of both autoantibodies may indicate a synergistic effect, where the combined immune attacks lead to an amplified release of ss-α3(IV)NC1, thereby defining a patient subgroup with exceptionally aggressive disease. Therefore, ANCA status is not a mere confounder to be adjusted for, but may be a key modifier that identifies a unique pathophysiological process within the anti-GBM disease spectrum. Another point to consider is that anti-GBM antibodies could potentially form immune complexes with ss-α3(IV)NC1, which could alter its clearance and half-life. The formation of immune complexes might theoretically protect the antigen from rapid degradation or clearance, thereby prolonging its circulation and contributing to an elevated ss-α3(IV)NC1 level. Additionally, the presence of anti-GBM antibodies could interfere with the ELISA assay by masking epitopes or competing with the detection antibodies, which could affect the accuracy of the measured ss-α3(IV)NC1 level. Further investigation of these hypotheses is required.

Correlation analysis revealed that ss-α3(IV)NC1 was positively correlated with crescent percentage and negatively correlated with normal glomeruli percentage. These two pathological indicators are hallmarks of anti-GBM disease and strongly correlated with kidney outcome (13, 17). Additionally, kidney survival analysis identified ss-α3(IV)NC1 as a risk factor for kidney prognosis, together with other known indicators. The multivariate Cox regression identified serum creatinine and normal glomeruli percentage as independent predictors of kidney outcome, whereas ss-α3(IV)NC1 level did not retain independent prognostic significance.

This finding suggests that the prognostic information conveyed by ss-α3(IV)NC1 level may be largely encompassed by these more direct indicators of kidney impairment. We hypothesize that this is due to the intrinsic pathophysiological link between the ss-α3(IV)NC1 level, the extent of the autoimmune attack, and the resultant functional and structural kidney damage. Consequently, the powerful prognostic value of serum creatinine (reflecting reserved kidney function) and kidney histology (reflecting structural integrity) appears to supersede that of the antigen itself. Taken together, we hypothesized that ss-α3(IV)NC1 level may be used as a potential serum marker of glomerular injury in patients with anti-GBM disease.

Circulating positive anti-GBM antibodies detected by commercial ELISA kits is the primary diagnosis criteria for anti-GBM disease. Although a spectrum of novel autoantigens has been uncovered in anti-GBM disease in recent years, such as α5(IV)NC1 (18, 19), peroxidasin (20), laminin 521 (21, 22), and perlecan (23), the discovered α3(IV)NC1 remains the most important defining feature of anti-GBM disease at present (24). Our current results suggested that not only positive anti-GBM antibodies could be detected in the circulation of patients with anti-GBM disease, but also α3(IV)NC1 level itself was abnormally elevated in a soluble form in a majority of patients. Indeed, previous studies showed that ss-α3(IV)NC1 (also known as tumstatin) could be present in humans under physiological conditions (8). The elevated ss-α3(IV)NC1 level might be attributed to the degradation of COL4A3 within the GBM hexamer by MMPs, especially MMP-9 (7). Although our study did not investigate the levels of MMP-9 in patients with anti-GBM disease, *in vivo* animal studies suggested that deletion of *Mmp9* alleviated the progression of anti-GBM nephritis (25). The mRNA expression of *Mmp9* was also found to be increased within the glomeruli and in parallel with the abnormal glomerular histology and injury in anti-GBM nephritis (26). These findings suggest a role for MMP-9 in the proteolysis of the GBM during anti-GBM disease, which may cause the release of α3(IV)NC1 into circulation. This hypothesis warrants and merits future detailed investigation. In addition, MMP-12 was recently shown to have a role in cleaving GBM components and thus exacerbated kidney pathology (12).

### Limitations

Limitations are acknowledged in our current study. First, owing to its observational nature, although a quantitative association between ss-α3(IV)NC1 level and the severity of kidney injury was found, we are currently unable to evaluate the causal relationship. Whether the loss of immune tolerance to α3(IV)NC1 could be attributed to elevated ss-α3(IV)NC1 remains unclear but merits future work-up. Second, statistical bias could not be avoided because the sample size was quite limited due the rarity of anti-GBM disease. We acknowledge that the sample size for patients with kidney biopsy data was limited to 41, which significantly restricted the statistical power of our pathological analyses and precluded a multivariate Cox analysis incorporating pathological indicators. This impedes a full understanding of prognostic factors. Third, a limitation of our study is the lack of data on precise disease duration prior to sampling and pre-existing co-morbid conditions. Consequently, we were unable to statistically adjust for these potential confounders, and their influence on ss-α3(IV)NC1 cannot be ruled out. Therefore, larger, more accurate, sample-based studies are needed in the future. Finally, we acknowledged the lack of relevant disease controls, such as active AAV patients with kidney injury, which helps to determine the specificity of elevated ss-α3(IV)NC1 level in anti-GBM disease.

## Conclusion

In conclusion, this is the first study to show that the ss-α3(IV)NC1 is abnormally elevated in patients with anti-GBM disease. An increased ss-α3(IV)NC1 level is associated with kidney injury and a poorer kidney outcome in patients with anti-GBM disease. Further research into the role of ss-α3(IV)NC1 could help to improve our understanding of the pathogenesis of anti-GBM disease.

## Data Availability

The original contributions presented in the study are included in the article/**Supplementary Material**. Further inquiries can be directed to the corresponding author.
