# Supplementary material for: Elevated serum soluble α3(IV)NC1 correlates with kidney injury and worse outcome in patients with anti-glomerular basement membrane disease
Source: Front Immunol. 2026 Feb 20;17:1728059. doi: 10.3389/fimmu.2026.1728059 (PMC12963254; doi:10.3389/fimmu.2026.1728059)
Supplement: Supplementary file 1 [file Table1.docx]

**Supplemental Table 1. Multivariate Cox regression analysis for clinical indicators to predict kidney survival.**

| Characteristic (*n* = 70) | HR (95% CI) | *P*-value |
| --- | --- | --- |
| Age (years) | 1.011 (0.988 to 1.034) | 0.361 |
| Gender (male) | 1.220 (0.630 to 2.362) | 0.555 |
| α3(IV)NC1 levels (ng/mL) | 1.024 (0.991 to 1.059) | 0.156 |
| Oligoanuria | 1.210 (0.558 to 2.625) | 0.629 |
| Serum creatinine (μmol/L) | 1.001 (1.001 to 1.002) | **< 0.001** |
| Initial need for RRT | 1.307 (0.578 to 2.951) | 0.520 |

RRT: renal replacement therapy; HR, hazard ratio; 95% CI, 95% confidence interval. Statistically significant differences were reported with a bold P-value.

**Supplemental Table 2. Multivariate Cox regression analysis for indicators to predict kidney survival.**

| Characteristic (*n* = 41) | HR (95% CI) | *P*-value |
| --- | --- | --- |
| Age (years) | 0.993 (0.960 to 1.028) | 0.690 |
| Gender (male) | 2.080 (0.829 to 5.217) | 0.119 |
| Crescents (%) | 1.002 (0.977 to 1.028) | 0.859 |
| Normal glomeruli (%) | 0.889 (0.827 to 0.956) | **0.002** |

HR, hazard ratio; 95% CI, 95% confidence interval. Statistically significant differences were reported with a bold P-value.
